# Clinicopathological Profile of *Salmonella Typhi* and *Paratyphi* Infections Presenting as Fever of Unknown Origin in a Tropical Country

**DOI:** 10.4084/MJHID.2015.021

**Published:** 2015-03-01

**Authors:** Nayyar Iqbal, Aneesh Basheer, Sudhagar Mookkappan, Anita Ramdas, Renu G’Boy Varghese, Somanath Padhi, Bhairappa Shrimanth, Saranya Chidambaram, S. Anandhalakshmi, Reba Kanungo

**Affiliations:** Pondicherry Institute of Medical Sciences, Ganapathichettikulam, Pondicherry, India

## Abstract

**Background:**

Enteric fever, a common infection in the tropics and endemic to India, often manifests as an acute febrile illness. However, presentation as fever of unknown origin (FUO) is not uncommon in tropical countries.

**Methods:**

We aim to describe the clinical, laboratory and pathological features of patients hospitalized with fever of unknown origin and diagnosed as enteric fever. All culture proven cases of enteric fever were analyzed retrospectively over a period of three years from January 2011 to December 2013.

**Results:**

Seven of 88 (8%) cases with enteric fever presented as FUO. Abdominal pain was the most common symptom besides fever. Relative bradycardia and splenomegaly were uncommon. Thrombocytopenia was the most common haematological abnormality while leucopenia was rare. Transaminase elevation was almost universal. *S. Typhi* and *S. Paratyphi* A were isolated from six cases and one case respectively. Yield of organisms from blood culture was superior to that of bone marrow aspirate. Multiple granulomas were identified in 4 out of 6 (67%) of the bone marrows studied, including that due to *S. Paratyphi* A and histiocytic hemophagocytosis was noted in two cases.

**Conclusion:**

FUO is a relatively common manifestation of enteric fever in the tropics. Clinical and laboratory features may be atypical in such cases, including absence of relative bradycardia, leucopenia, and presence of thrombocytopenia, bicytopenia or pancytopenia. In addition, in endemic countries, enteric fever should be considered as a differential diagnosis, next to tuberculosis, in the evaluation of bone marrow granulomas in cases with FUO and culture correlation should be mandatory.

## Introduction

Enteric fever is associated with high morbidity in the tropics. Although mortality has reduced due to antibiotic treatment, it is still a cause of concern due to increasing antibiotic resistance. The disease still remains endemic in South Asia including India. An incidence of around 214.2/100 000 cases per year is reported from India.^1^ It is higher among children under five years of age as compared to adults.^2^ Mortality due to enteric fever depends on time taken to diagnose and institute antibiotic treatment. Mortality is less than 1% if treatment is started before onset of complications, and increases to around 15% if treatment is started after onset of complications.^3^ Enteric fever is confirmed by blood culture. However, this method has variable sensitivity depending on the duration of fever and prior antibiotic therapy. It is most sensitive in the early part of infection (within seven days). The rate falls with increasing duration of fever. Widal test, a commonly used serological test in India has very low specificity and is less reliable. Apart from presenting as FUO, rare presentations like disseminated intravascular coagulation, haemophagocytic syndrome, splenic abscess, granulomas, pancreatitis, and hepatitis have also been reported.^4^ A study from India showed that *S. Typhi* infection is more likely to manifest as fever of unknown origin when compared to *S. Paratyphi*.^5^

## Materials and Methods

Medical records were reviewed to retrospectively identify all cases diagnosed as enteric fever, typhoid fever, paratyphoid fever, *Salmonella Typhi or Paratyphi* (A, B or C) infection (hereafter referred to as enteric fever in this article). Between January 2011 and December 2013, there were 98 case records with a diagnosis of enteric fever. Seven cases were excluded as the diagnosis was based on clinical features and response to ceftriaxone. Of the remaining 91 cases, clinical and laboratory data were not fully available in 3 cases. Finally, 88 cases were included for review in which the diagnosis of enteric fever was confirmed by growth of *S. Typhi or S. Paratyphi* (A, B or C) in blood or bone marrow culture or both. Among these, seven patients fulfilled criteria for classic FUO, defined as temperature of more than 101°F for more than three weeks duration without reaching a diagnosis even after three outpatient visits, or three days of in hospital stay, or one week of intelligent and invasive ambulatory investigations.

We analyzed clinical and laboratory data of these 7 cases, including blood counts, biochemical parameters, bone marrow and imaging studies. The clinical and laboratory data were analyzed using descriptive statistics (mean, median and standard deviation).

## Results

Between January 2011 and December 2013, seven out of 88 cases (8%) of enteric fever presented as classic FUO. These included five males and two females with an age range of 19 to 45 years. All 7 cases had fever for more than three weeks, with a median duration of 26 days at presentation to the hospital. The other significant symptoms were abdominal pain noted in 5 (71%) cases, headache in 4 (57%) cases followed by diarrhea in 3 out of 7 cases; 43%). Relative bradycardia was noted in one of the 7 (14%) cases. Table 1 summarizes the frequency of various symptoms and signs in the seven cases.

Isolated thrombocytopenia was the most common haematological abnormality in these patients (3/7, 43% of cases). The mean platelet count at admission was 85 × 10^9^ cells/L (Ref.: 150–450 × 10^9^/L). Isolated anemia was uncommon (1 out of 7 cases) and isolated leucopenia was not observed in any case. Bicytopenia was found in 1 (14%) case, while pancytopenia was seen in 2 (29%) cases. The mean blood counts are summarized in Table 2.

Urine examination and renal functions were normal in all except one case who had mild azotemia and significant pus cells in urine. Liver functions were abnormal in 6 out of 7 (86%) patients (Table 2). In all these cases, aspartate aminotransferase (AST) and alanine aminotransferase (ALT) were elevated with the former being significantly higher than the latter (AST/ALT > 1). Alkaline phosphatase levels were elevated in only one patient, who also had direct hyperbilirubinemia and elevated transaminases.

Gall bladder wall edema was noted on ultrasound abdomen in 2 cases. Computerised tomography (CT) of abdomen was done in two patients. It revealed caecal and terminal ileal wall thickening with enlarged mesenteric lymph nodes in one case and splenic infarction and pancreatitis in another. One case who presented with meningeal signs underwent CT brain that revealed cerebral edema.

Diagnosis of enteric fever was established in all seven cases within a median duration of 6 days of hospitalization. Blood culture grew *S. Typhi* in four cases (57%) and *S. Paratyphi* A in one case (14%). In the remaining two (29%) cases, diagnosis was made by culture of *S. Typhi* from the bone marrow (Table 3). Overall, bone marrow biopsy and aspiration was done in 6 of the 7 cases, and *S. Typhi* was cultured from marrow in 2 cases and *S. Paratyphi* A in one. Multiple non-caseating granulomas were observed in four (67%) of these marrows **(**Figure 1A and B). In one case, bone marrow was reported as reactive, and histiocytic haemophagocytosis was noted in two.

Prior to admission in our institution, 3 of the seven patients had received oral cefixime and 2 received injection amikacin while the remaining 2 were given both oral ofloxacin and injection ceftriaxone from elsewhere. However, data on duration of treatment taken from outside hospitals were not available. None of the patients had received steroids. All patients were treated at our institution with ceftriaxone injection. They responded with defervesence within a mean period of 4 days, except one of the two cases with histiocytic haemophagocytosis who succumbed on the fourth day of hospitalization, despite appropriate antibiotic therapy.

## Discussion

With early diagnosis and specific therapy complications due to enteric infections have come down in the recent past. However, presentation as fever of unknown origin may pose a diagnostic dilemma. Enteric fever presenting as FUO is not uncommon in India due to endemicity in this part of the world.^3^,^6^,^7^ In our study out of 88 only 7 (8%) patients were found to fulfill criteria for FUO. Prior to admission to our hospital, they were all investigated elsewhere but without arriving at a definite diagnosis.

Relative bradycardia at the peak of fever is a well described feature of enteric fever. Neopane and colleagues proposed relative bradycardia as a major criterion with a high diagnostic accuracy in clinical diagnosis of enteric fever, especially in tropics.^8^,^9^ In a study of 35 patients with enteric fever from Taiwan, relative bradycardia was noted in 25% of cases. FUO was the most common presentation of enteric fever in the above series.^10^ However, in our cases relative bradycardia was relatively uncommon (1/7, 14%); whether this is peculiar to enteric fever presenting as FUO can only be answered by large prospective studies. Gastrointestinal bleed occurs in 10–20% of cases with enteric fever. In our study, 28% of cases had gastrointestinal bleed in the form of malena and/or hematochezia. One case had aseptic meningitis with sixth nerve palsy, a finding that has rarely been described in literature.^6^ Hepatomegaly and splenomegaly were appreciated in one case each; again a significantly lower frequency compared to other studies. Splenomegaly, in particular, seems to have a higher predictive value in the clinical diagnosis of enteric fever.^11^ Haematological abnormalities were relatively common in our cases, thrombocytopenia and anemia being the predominant manifestations. Leucopenia, the classic laboratory finding described in enteric fever was encountered in only one patient, and the mean total leucocyte count was in the normal range. In addition none of the cases had isolated leucopenia. Pancytopenia, a relatively infrequent manifestation of enteric fever was noted in two of our patients. All these indicate that haematologic manifestations of enteric fever may not follow well described prototypal patterns in the tropics, the reason for which needs to be studied. In fact, a recent survey found that, although leucopenia was a significant finding in enteric fever, thrombocytopenia was highly predictive of the disease along with relative bradycardia, splenomegaly, rose spots and elevated AST.^11^

6 out of 7 (86%) cases in our series had elevated liver enzymes (AST and ALT). We observed that all the cases of transaminitis had greater elevations of AST compared to ALT, with a mean AST to ALT ratio of 1.8. These findings are similar to those of studies by A Ahmad et al., Herdiman T Pohan and Morgenstern R et al.^12^,^13^,^14^ According to a study by Khossla et al^15^ typhoid hepatitis may be diagnosed if a patient fulfilled three or more of the following criteria: hepatomegaly, jaundice, biochemical abnormalities (increased bilirubin, increased AST/ALT, deranged prothrombin time) and abnormal histopathology. Applying these criteria only 1 (14%) patient had typhoid hepatitis. Morgenstern R et al. found that increased AST and ALT were noted in second or third week of illness, an observation that was observed in our study as well. The cause for abnormal liver function in enteric fever is still poorly understood. It is believed to be endotoxin related or due to secondary immune mechanism.^16^

The most commonly described CT abnormalities in enteric fever include mesenteric lymphadenopathy and splenomegaly followed by circumferential bowel wall thickening.^17^ These findings were present in one patient in our study.

Acute pancreatitis was observed in one patient. Acute pancreatitis has been infrequently reported as a complication of enteric fever.^18^ The cause for acute pancreatitis is thought to be same as hepatitis. This patient also had splenic infarction. Although Ali Mert et al.^19^ have reported a case of typhoid with splenic granuloma, splenic infarct is distinctly unreported in enteric fever.

The sensitivity of blood culture in isolation of *Salmonella* varies from 40 to 80% as compared to bone marrow culture where it is up to 90%. The reason for bone marrow culture being considered superior is that prior antibiotic use does not interfere with isolation of S. Typhi from marrow unlike blood culture.^3^ The most common organism isolated in our study was S. Typhi (6 out of 7 cases, 86%). Bone marrow aspiration, biopsy, and culture were done in six patients as part of work up for FUO. The organisms (S. Typhi and S. Paratyphi A) were isolated from bone marrow in 3 cases (43%). Yield of salmonella from blood was superior to that from bone marrow aspirate. We noted 71% positivity in the blood cultures as against 43% from the marrow. Shin et al.^20^ reported a culture positivity of 25 % from bone marrow and 62% from blood in their series. In another study from Karachi, S. Typhi was isolated from bone marrow in all cases of enteric fever that presented as FUO whereas it was isolated from blood only in 66% of the cases; the authors concluded that bone marrow cultures may be superior in diagnosing typhoid fever when blood cultures are sterile.^21^ In contrast, we noted a better yield from repeat blood cultures than from marrows of patients whose initial workup for FUO elsewhere was unrewarding. Moreover, all our patients had already received one or more antibiotics from local facilities. Hence, we believe that despite prior antibiotic therapy and initial negative blood cultures, it may be a good practice to perform repeat blood cultures rather than ordering a bone marrow culture upfront. Although our findings suggest that bone marrow cultures may not be superior to blood cultures in diagnosis of enteric fever presenting as FUO in tropics, prospective studies with larger numbers are needed to confirm this.

Four out of six (67%) cases had multiple granulomas in the marrow. In our study, we found 75 % of ill-defined and 25% of well-defined granulomas in the marrow. This includes a unique case of bone marrow granuloma with phagocytosed debris in a culture proven case of S. Paratyphi A. To the best of our knowledge, this is only the second such case reported in world literature after the case by Lee et al..^22^ Bone marrow granulomas due to enteric fever were rarely reported before 1985 when Lee et al.^23^ described granulomas in their large series of 27 patients. Shin et al.^20^ also reported granulomas in 8 cases of enteric fever and characterized them according to disease stage. In both studies, well-formed granulomas were noted in 57% and 50% of cases respectively while ill-defined granulomas were observed in 43% and 50 % of cases respectively. Shin et al. reported that histiocytic proliferation was more common in early stage, ill-defined histiocytic granulomas with phagocytes debris present in the proliferative stage. Furthemore, well-formed histiocytic and/or epithelioid granulomas with erythrophagocytosis were often seen during the lysis phase (corresponding to third and fourth week) of the disease. Hence, the high frequency of bone marrow granulomas in our patients may be due to the fact that all of them were in the fourth week of illness, corresponding to the late/lysis stages. This finding may have a significant clinical implication as extrapulmonary and/or disseminated tuberculosis, being the commonest etiology for FUO in countries like India is often diagnosed by the presence of granulomas in the marrow. Hence, morphologically distinguishing tubercular granuloma from that of enteric fever is essential; and in the absence of culture correlation could be challenging. Overall, tubercular granulomas are characterized by ill formed to well defined aggregates of epithelioid histiocytes, caseous necrosis, surrounding lymphoplasmacytic infiltrate, and/or the hall mark Langhan giant cell.^24^ In contrast, as described in the literature,^19^,^20^,^22^,^23^ ill formed histiocytic and/or epithelioid granulomas are more frequent in enteric fever. Histiocytic cells with phagocytosed debris (so called “typhoid cells” in bone marrow aspirate smears) with or without erythrophagocytosis may serve as important features of typhoidal granulomas; especially if they are well formed. Haemophagocytosis was evident in the marrow of two patients, who fulfilled the HLH-2004 criteria for haemopahgocytic lymphohistiocytosis (HLH). This syndrome has infrequently been reported in typhoid fever.^25^ While one patient improved with antibiotic therapy, the other expired from multiorgan failure.

Six out 7 (86%) cases responded to treatment with injection ceftriaxone. A review of randomized control trials concluded that azithromycin may perform better than ceftriaxone in enteric fever.^26^ A recent study from India reported resistance to third generation cephalosporins among 2% of isolates of Salmonella enterica, which was lower compared to resistance to fluoroquinolones and azithromycin.^27^ Ciprofloxacin resistance was noted in 8% of the isolates in the present study. Although, we have noticed a trend towards increase in the minimum inhibitory concentration (MIC) of ceftriaxone for S. Typhi (unpublished data from our institution), we did not find any true resistance among our cases. For this reason, azithromycin was not used in any of our cases. We propose that ceftriaxone should be considered as the first line antibiotic for enteric fever in this part of the world, although with close monitoring of the MIC.

## Conclusions

Enteric fever is a relatively frequent cause of fever of unknown origin in the tropics. When it presents as FUO, clinical and laboratory manifestations may not be typical, especially features such as relative bradycardia, leucopenia, and splenomegaly. Isolation from bone marrow may not always be superior to that from blood in these cases, and it may be worthwhile performing repeated blood cultures even in the face of prior negative cultures and administration of antibiotics. Bone marrow evaluation is as important as culture and must be meticulously examined for granulomas and phagocytosed debris. This bone marrow picture may be an increasingly recognized feature of enteric fever presenting with prolonged pyrexia and needs to be differentiated from the more common granulomas of disseminated tuberculosis. Larger prospective studies are required to establish distinct clinico-pathologic patterns of enteric fever presenting as fever of unknown origin.

## Figures and Tables

**Figure 1A:**
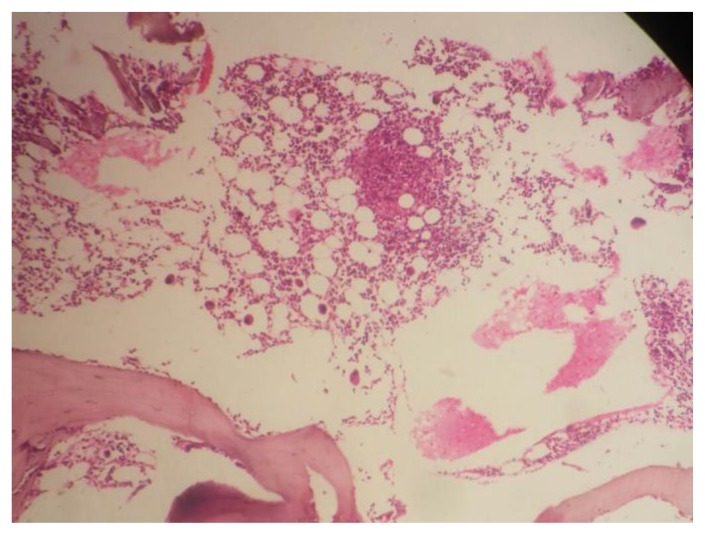
Bone marrow trephine biopsy showing focal granuloma (H&E, 100X)

**Figure 1B:**
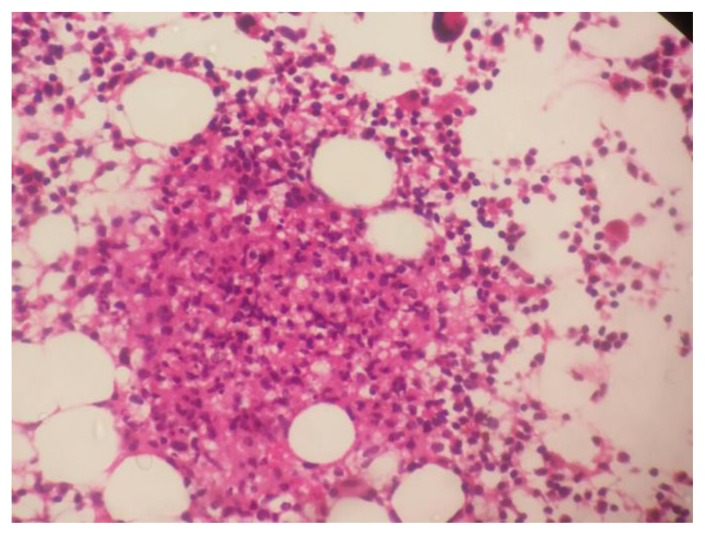
Bone marrow trephine biopsy showing non-caseating epithelioid cell granuloma (H&E, 400X)

**Table 1:** Clinical features of enteric fever patients who presented as FUO (n = 7).

Symptom/sign	Number of patients (%)
Fever	7 (100)
Abdominal pain	5 (71)
Headache	4 (57)
Diarrhea	3 (43)
Constipation	1 (14)
Vomiting	2 (29)
Neck pain	1 (14)
GI bleed	2 (29)
Jaundice	1 (14)
Pallor	3 (43)
Signs of meningeal irritation (neck rigidity/Kernig’s sign)	1 (14)
Hepatomegaly	1 (14)
Splenomegaly	1 (14)
Lymphadenopathy	1 (14)
Relative bradycardia	1 (14)

**Table 2:** Mean blood parameters in enteric fever presenting as PUO (n = 7)

Blood parameter	Mean value	Standard deviation
Haemoglobin (g/dl)	10.6	2.0
Total leucocyte count (x 10^9^/L)	4.9	2.0
Platelet count (x10^9^/L)	85	58
AST (U/L)	164	101
ALT (U/L)	88	72
ALP (U/L)	140	113
Serum albumin (g/dl)	3.3	0.6
Blood urea (mg/dl)	27	13.7
Serum creatinine (mg/dl)	0.6	0.13

**Table 3:** Isolates from blood and marrow in patients with enteric fever who presented as PUO. (n = 7)

Organism isolated	Specimen	Number (%)
*S. typhi*	*Blood*	4 (57)
	*Bone marrow*	2 (29)
*S. paratyphi*	*Blood & bone marrow*	1 (14)
